# Assessing farmland suitability for agricultural machinery in land consolidation schemes in hilly terrain in China: A machine learning approach

**DOI:** 10.3389/fpls.2023.1084886

**Published:** 2023-03-06

**Authors:** Heng Yang, Wenqiu Ma, Tongxin Liu, Wenqing Li

**Affiliations:** ^1^ College of Engineering, China Agricultural University, Beijing, China; ^2^ Key Laboratory of Land Consolidation and Rehabilitation, Land Consolidation and Rehabilitation Center, Ministry of Natural Resources, Beijing, China

**Keywords:** farmland consolidation suitable for agricultural machinery, suitability assessment, potential of farmland productivity, machine learning approach (MLA), zoning, hilly terrain

## Abstract

Identifying available farmland suitable for agricultural machinery is the most promising way of optimizing agricultural production and increasing agricultural mechanization. Farmland consolidation suitable for agricultural machinery (FCAM) is implemented as an effective tool for increasing sustainable production and mechanized agriculture. By using the machine learning approach, this study assesses the suitability of farmland for agricultural machinery in land consolidation schemes based on four parameters, i.e., natural resource endowment, accessibility of agricultural machinery, socioeconomic level, and ecological limitations. And based on “suitability” and “potential improvement in farmland productivity”, we classified land into four zones: the priority consolidation zone, the moderate consolidation zone, the comprehensive consolidation zone, and the reserve consolidation zone. The results showed that most of the farmland (76.41%) was either basically or moderately suitable for FCAM. Although slope was often an indicator that land was suitable for agricultural machinery, other factors, such as the inferior accessibility of tractor roads, continuous depopulation, and ecological fragility, contributed greatly to reducing the overall suitability of land for FCAM. Moreover, it was estimated that the potential productivity of farmland would be increased by 720.8 kg/ha if FCAM were implemented. Four zones constituted a useful basis for determining the implementation sequence and differentiating strategies for FCAM schemes. Consequently, this zoning has been an effective solution for implementing FCAM schemes. However, the successful implementation of FCAM schemes, and the achievement a modern and sustainable agriculture system, will require some additional strategies, such as strengthening farmland ecosystem protection and promoting R&D into agricultural machinery suitable for hilly terrain, as well as more financial support.

## 1 Introduction

Since the 20th century, continuing population growth, accompanied by rapid urbanization and industrialization has intensified the need to increase food production on farmland (Wang et al., 2021). Agricultural machinery has become an increasingly important tool to ensure a sufficient global supply of food. Identifying the available farmland suitable for agricultural machinery is believed to an effective sustainable development method, because mechanized agriculture is a strategy that is fundamental to efforts to alleviate poverty and food security by greatly increasing grain productivity. Farmland consolidation suitable for agricultural machinery (FCAM) is an effective tool for increasing sustainable production through the implementation of mechanized agriculture (Zhong et al., 2020).

FCAM is derived from farmland consolidation, the aim of which, based on principles modern of agriculture, is to increase the productivity and profitability of farmland that is small and fragmented parcels of farmland for various reasons (Nilsson, 2019; Basista and Balawejder, 2020; Uyan et al., 2020; Duan et al., 2021; Beyer et al., 2022). The aim of FCAM is to identify the most suitable places for the operation of agricultural machinery (Collins et al., 2001; Duan et al., 2021; Janus and Ertunç, 2021; Beyer et al., 2022). In many countries (e.g., Japan and South Korea), FCAM is used as a land management tool to eliminate the adverse effects of land fragmentation and to improve and promote agricultural mechanization (Callesen et al., 2022; Washizu and Nakano, 2022). The rules and regulations for FCAM projects vary from country to country (Janus and Ertunç, 2022). However, in every country that implements FCAM, it is essential to assess the suitability of farmland plots in the project area (Jiang et al., 2017; Giri et al., 2019; Dias et al., 2021; Jiang et al., 2022).

The identification of farmland suitable for agricultural machinery in land consolidation schemes is critical for improving agricultural production as well as optimizing land use. Recently, this process has also attracted the attention of the scientific community. Some scholars have stated that assessments of farmland suitability in land consolidation schemes can be characterized as a decision problem involving several factors that quantify the relative importance of the criteria to be considered in the study (Morales and de Vries, 2021). There are many examples of assessments of farmland suitability (Akpoti et al., 2019; Mazahreh et al., 2019; Pilevar et al., 2020), and a variety of variables, including the physical attributes of the land, soil properties, agricultural infrastructure (e.g., irrigation and drainage), and socioeconomic factors, have been incorporated into the suitability assessment process (Vasu et al., 2018). In addition, some scholars have used analytical methods to determine if a piece of farmland is suitable for agricultural machinery (Mazahreh et al., 2019; Al-Taani et al., 2021). The methods include qualitative description, spatial analysis, and other modern techniques. For instance, there is a rich body of literature describing farmland suitability using analytic hierarchical processes (AHPs), dynamic system models, and other multicriteria analysis (MCA) (Seyedmohammadi et al., 2019; Pilevar et al., 2020; Morales and de Vries, 2021). Advancements in spatial analysis methods have led scholars to prefer the use of remote sensing (RS) and geographic information systems (GIS) to assess the suitability of farmland (AbdelRahman et al., 2016; Fu et al., 2019; Habibie et al., 2019; Kumar et al., 2021), which in turn has led to the inclusion of an extensive range of variables in the process of analyzing suitability. Recently, the application of artificial intelligence (AI) has received a great deal of attention in land use management and has brought about revolutionary changes in assessing farmland suitability (Nguyen et al., 2020; Xu et al., 2021). In this sense, assessing the suitability of FCAM has become a multidimensional and multidisciplinary process that integrates numerous aspects in which different criteria are weighted.

In China, agricultural production in hilly terrain is a critical issue because, although farmland accounts for over 30% of such areas, and 50% of residents in these areas are engaged in agricultural labor, the level of agricultural mechanization is below 50%, which is far below the national level (72.03% in 2021). Existing studies have confirmed that this considerable gap in agricultural mechanization can be attributed to inferior farmland conditions (e.g., scattered plots, steep slopes, and low-quality soil), which render farmland unsuitable for mechanized agriculture. Therefore, implementing FCAM (which entails such processes as enlarging plots, land leveling, soil improvement, and ecological protection) in hilly terrain is indispensable to promoting mechanized agriculture (Yin et al., 2022). Moreover, the use of land suitability analysis in FCAM can contribute to the sustainable development of rural marginal areas (Zhang et al., 2019a) by indicating the most suitable farmland plots, which has both economic and ecological benefits, increasing crop yield and improving soil properties, contributing to a long-term purpose—achieving modern agriculture and food security (Wessels et al., 2003; Fan et al., 2018).

In presenting analyses, however, studies have been criticized for lacking a deep understanding of FCAM schemes because these assessments of farmland suitability and farmland consolidation focus merely on the physical attributes of farmland. Detailed analyses of farmland suitability for agricultural machinery are still rare. More systematic assessments reflecting the characteristics of agricultural machinery as well as farmland ecological limitations are needed to better understand the suitability and implications of FCAM schemes. In addition, although there is evidence of the methods involved in the qualitative description, spatial analysis, and other modern techniques, it is necessary to develop a precise and scientifically proven method for weighting the suitability criteria that is compatible with FCAM.

This study aims to assess the suitability of farmland for agricultural machinery in land consolidation schemes using the most appropriate method—the machine learning approach. It also attempts to estimate the potential of farmland productivity after the implementation of FCAM schemes. The remainder of this paper is structured as follows: section 2 details the source of the sample data and provides a descriptive analysis of machine learning approaches; section 3 presents the results of the study; in section 4 we discuss the implications of the study; and, finally, in section 5 we draw conclusions.

## 2 Materials and methods

### 2.1 Study area

**Figure 1:**
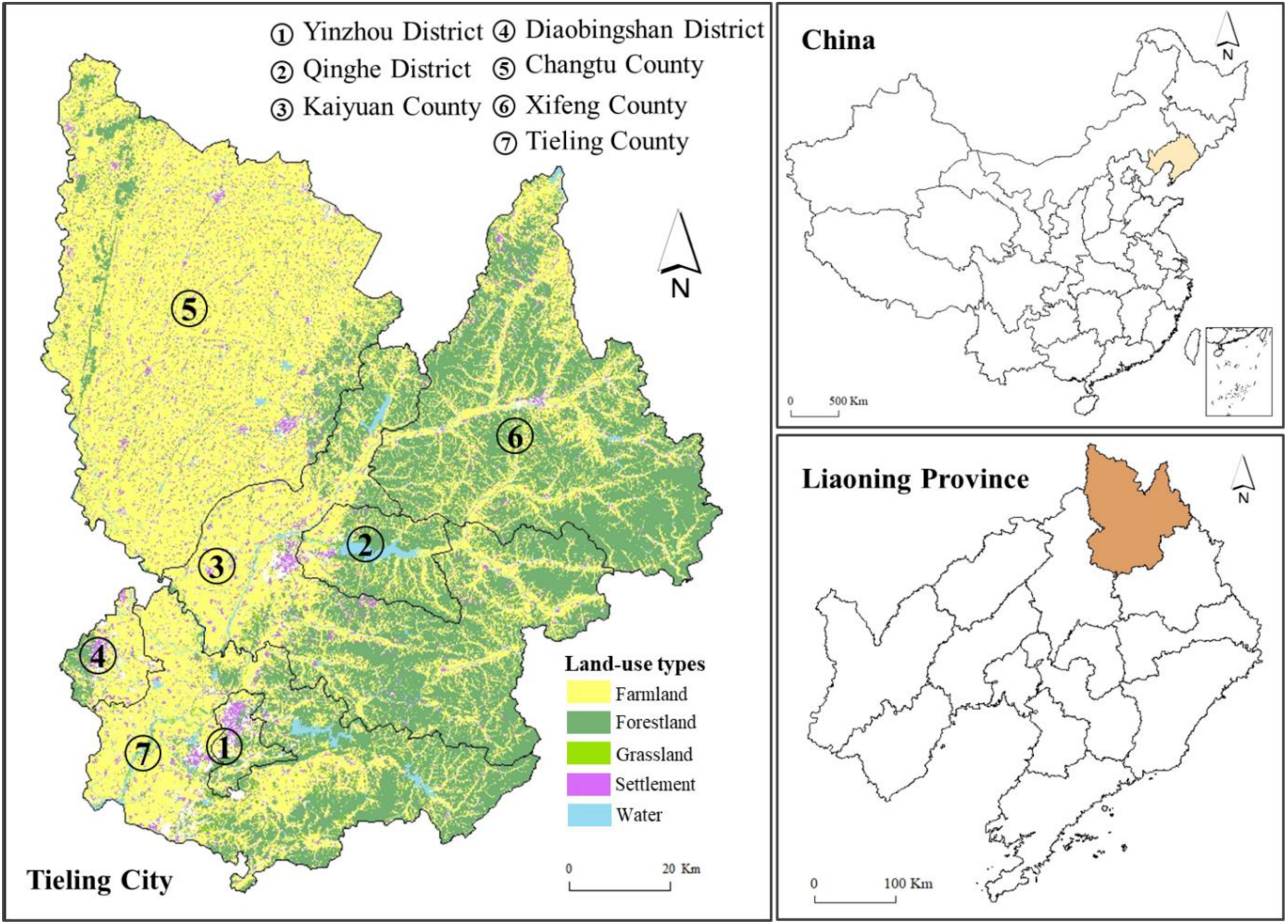
Study area.

Tieling City (41°59′N–43°29′N, 123°27′E–125°06′E) is located in northern Liaoning Province, northeastern China. It comprises two districts, five counties, 89 towns, and 1,290 villages. In 2021, the city occupied an area of approximately 1.3 million ha and had a population of 2.827 million. The altitude of the city ranges from 50 m to 878 m above sea level (**Figure 1**). This indicates that the topography of the area ranges from lowland plain city to highly rugged and elevated hilly terrains. Because of the nature of these elevated and hilly areas, the morphology of the study area is quite complex: the edges of the eastern and northern parts of the city are characterized by steep slopes, whereas, toward the center and the south, the morphology is characterized by quite gentle slopes, although there are still some steep slopes along the river courses and on hilly terrain. Hilly terrain accounts for 40% of the total area.

It is well known that economy of Tieling City is based on agricultural production. The major crops grown are corn and rice, and its cropping system is growing only one crop on the same field in a year. Influenced by a temperate continental monsoon climate, the annual average temperature and rainfall are 7.9°C and 600 mm, respectively. The city’s climate, as well as the available soil types of black, meadow, and rice soil, make it an ideal place for agriculture. Inn 2019, farmland area was 699.2 thousand ha, accounting for 53.8% of the city’s total land area. However, because of the long-term pursuit of “high-yield but low-input” crops in Tieling City’s agricultural production, land degradation has become an urgent problem, together with the complex and irregular topography of its hilly terrain. Consequently, it is necessary to implement FCAM schemes to improve Tieling City’s agricultural productivity.

### 2.2 Data

In this study, suitable lands for surface irrigation were identified using GIS and RS combined with a machine learning approach. To achieve this, the datasets are listed as follows.

#### 2.2.1 Land use data

Land use data were obtained from satellite image classification. For this study, Landsat 8 satellite images were used for classification. The land use data were classified into 25 categories, which were subsequently grouped into six classes: cropland, woodland, grassland, water body, built-up area, and unused land. From these data, we extracted data relating to farmland, agricultural roads, rivers, settlements, etc. The data were used to analyze the shape index of a plot, farmland area, accessibility of roads, irrigation rate, distance from agricultural machinery service cooperation, and distance from roads using ArcGIS 10.6 (Esri, Redlands, CA, USA).

#### 2.2.2 Digital elevation model

Elevation data with 30 m spatial resolution came from the SRTM1 V3.0 dataset (https://earthdata.nasa.gov/). These data were used to depict topographic features, including slope and altitude, by using slope analysis and aspect analysis in ArcGIS 10.6.

#### 2.2.3 Normalized difference vegetation index

The vegetation coverage of farmland was calculated using the normalized vegetation index (NDVI). The NDVI dataset was obtained from Landsat 8 satellite images. The dataset was provided by the National Ecosystem Science Data Center, National Science & Technology Infrastructure of China (http://www.nesdc.org.cn).

#### 2.2.4 Soil data

Soil data were used to assess the suitability of ecological limitations for the FCAM, which included soil pH, soil organic carbon, and soil microorganisms. A soil dataset with 250 m spatial resolution was provided by The World Soil Information Service (WoSIS, https://soilgrids.org/).

#### 2.2.5 Potential of farmland productivity

These data were provided by the Resource and Environment Science and Data Center (https://www.resdc.cn/). These data were estimated by Xu et al. (2010) to obtain the potential of farmland productivity by considering various factors. We use these data to estimate the potential of farmland productivity after the implementation of FCAM.

### 2.3 Methods

#### 2.3.1 Indicator system for assessing the suitability of FCAM

This research assessed the suitability of FCAM by establishing an indicator system. Based on the literature and indicator selection rules, i.e., integration, independence, diversity, and feasibility (Zhang et al.,2018; Pašakarnis et al., 2021), we selected 15 indicators from four dimensions, i.e., natural resource endowment, accessibility of agricultural machinery, socioeconomic level, and ecological limitations (see **Table 1**).

**Table 1:** Description of the indicators assessing the suitability of FCAM.

Dimensions	Indicators	Formula	Explanation	Direction
Natural resource endowment (0.19)	Slope (N_1_) (0.11)	N1=∑i=1NSiN	*S_i_* = Slope of the farmland plot *i* *N *= Total number of farmland plots	–
	Elevation (N_2_) (0.04)	N2=∑i=1NEiN	*E_i _* = Elevation of the farmland plot *i* *N *= Total number of farmland plots	–
	Soil thickness (N_3_) (0.03)	N3=∑i=1NHiN	*H_i _* = Soil thickness of the farmland plot *i* *N *= Total number of farmland plots	–
Accessibility of agricultural machinery (0.42)	Shape index of farmland (A_1_) (0.08)	A1=Pi4Ai	*P_i_* = Perimeter of the farmland plot *i* *A_i_* = Area of the farmland plot *i*	+
	Aggregation index of farmland (A_2_) (0.04)	A2=[1+∑i=1NPiln(Pi)2lnN]×100	*P_i_*_ _= Perimeter of the farmland plot *i* *N *= Total number of farmland plots	+
	Connectivity of farmland (A_3_) (0.08)	A3=∑i=1NAiN	*A_i_*_ _= Area of farmland plot *i* *N *= Total number of farmland plots	+
	Surface barriers (A_4_) (0.09)	A4=PsAt	*P_S_* = Perimeter of the surface barriers *A_t _* = Total area of farmland	+
	Accessibility of tractor roads (A_5_) (0.1)	A5=ArAt	*A_r _* = Area of roads used by agricultural machinery *A_t _* = Total area of farmland	+
	Distance to agricultural machinery service cooperation (A_6_) (0.02)	A6=∑i=1NDiN	*D_i_*_ _= distance of farmland plot *i* to agricultural machinery service cooperation *N *= Total number of farmland plots	–
Socioeconomic level (0.26)	Proportion of GDP in agricultural sector (*S_1_*) (0.09)	S1=GDPaGDPt	*G_a _* = GDP in agricultural sector *G_t_* = Total GDP	+
	Proportion of labor in agricultural sector (*S_2_*) (0.09)	S2=PaPt	*P_a_* = The population engaged in agricultural sector *P_t_* = Total working-age population	+
	Household income (*S_3_*) (0.08)	S3=INgn	*IN_G _* = Gross income from the household-life expenditures *N* = Total number of households	+
Ecological limitations (0.14)	Soil organic matter (E_1_) (0.06)	E1=∑i=1NOiN	*O_i _* = Soil organic matter content of the farmland plot *i* *N *= Total number of farmland plots	+
	Soil pH (E_2_) (0.02)	E2=∑i=1NpHiN	*pH_i _* = Soil pH value of the farmland plot *i* *N *= Total number of farmland plots	+
	Vegetation coverage (E_3_) (0.06)	E3=∑i=1NNDVIiN	*NDVI_i _* = NDVI of the farmland plot *i* *N *= Total number of farmland plots	–

GDP, gross domestic product.

Weights are shown below the indicators.

##### 2.3.1.1 Natural resource endowment

The natural resource endowment dimension comprised three indicators: slope, elevation, and soil thickness. Slope and altitude were selected to illustrate the influence of topography on FCAM, as these have been found to be factors that are important in determining suitability for FCAM. Slope is particularly important in determining suitability for FCAM because it is associated with farmland use patterns (Yao et al., 2021). In particular, a slope greater than 25° is considered unsuitable for agricultural production, because along with the increasing elevation come changes in precipitation and temperature, which limit agricultural development (Zhao and Zhang, 2022; Zhu et al., 2022). In addition, soil thickness was used to indicate soil fertility; however, as noted by Gardi et al. (2016), excessive soil thickness makes land unsuitable for agricultural machinery operation.

##### 2.3.1.2 Accessibility of agricultural machinery

We selected six indicators to demonstrate the accessibility of agricultural machinery. Existing studies have indicated that the physical attributes of farmland plots, as well as the convenience of agricultural machinery operation, greatly affect the accessibility of agricultural machinery (Akıncı et al., 2013; Liu et al., 2021; Liao et al., 2022). Therefore, the effects of shape index of farmland, aggregation index of farmland, connectivity of farmland, and surface barriers demonstrate the influence of land morphology on the suitability for FCAM. In most cases, the continuity and integrity of plots facilitates land consolidation. (Jiang et al., 2017; Li and Chen, 2020). In addition, we selected two indicators (i.e., accessibility of tractor roads and distance to agricultural machinery service cooperation) to demonstrate the convenience of operation. Notably, the distance to agricultural machinery service cooperation indicated the possibility of obtaining agricultural machinery services (Du et al., 2018).

##### 2.3.1.3 Socioeconomic level

Industry and population are two critical influencing factors for socioeconomic development (Mitter et al., 2020; Callesen et al., 2022). A high level of socioeconomic development can facilitate the implementation of FCAM (Jiang et al., 2015; Mugiyo et al., 2021). Therefore, we selected three indicators, i.e., proportion of GDP in the agricultural sector, proportion of labor in the agricultural sector, and household income, to show the socioeconomic level.

##### 2.3.1.4 Ecological limitations

In presenting analyses, the ecological effects of FCAM have been discussed by several scholars (Abubakari et al., 2016; Huang et al., 2022; Lu et al., 2022). On the one hand, FCAM can affect aspects of soil quality (such as soil organic matter and soil pH) and even result in land degradation (Keller et al., 2019; Wu et al., 2019; Paul et al., 2020; Zhang et al., 2022). More impracticable, impracticable FCAM schemes, such as building terraces, can reduce vegetation coverage and cause ecological fragility (Zhang et al., 2019b). All things considered, there are ecological limitations for FCAM, and we selected three indicators to illustrate these limitations.

#### 2.3.2 Weighting analysis based on the machine learning approach

**Figure 2:**
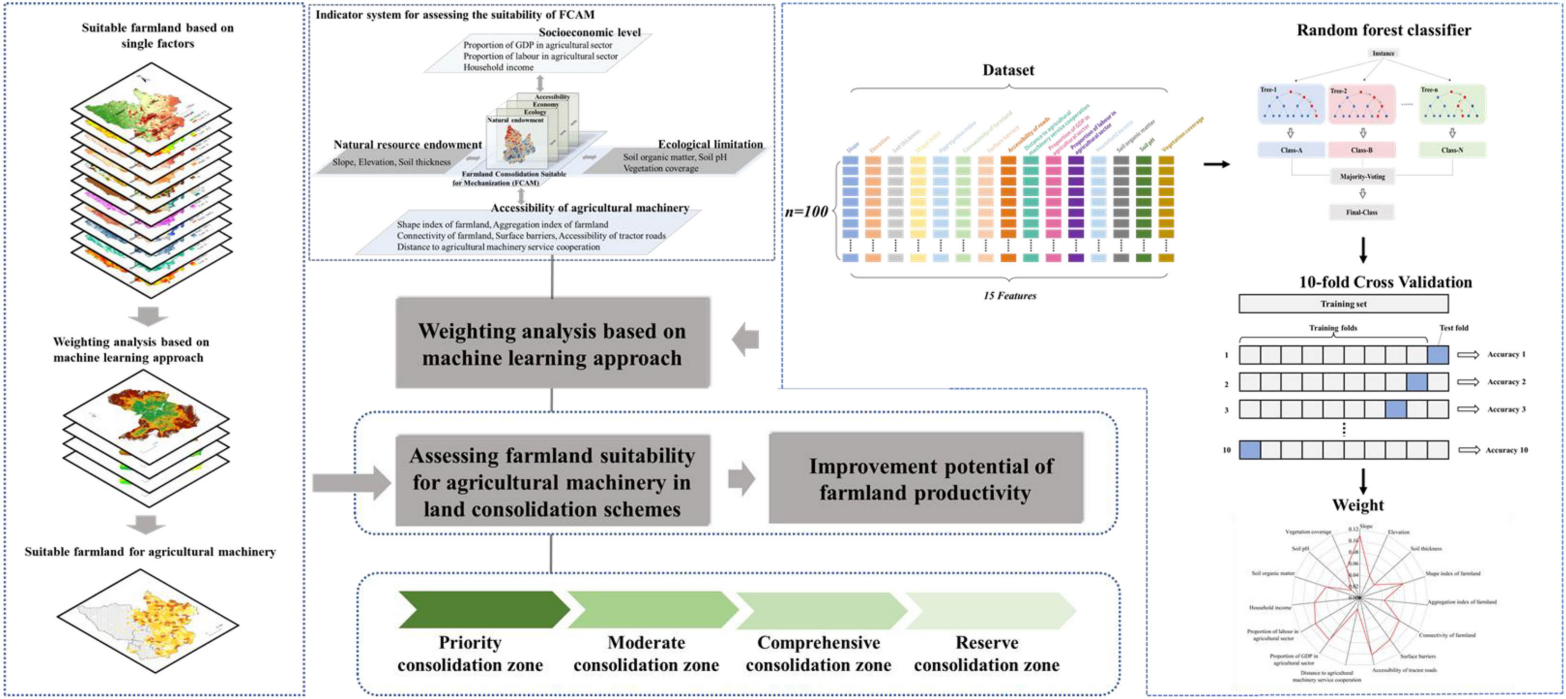
Methods for the assessment of the suitability of FCAM. FCAM, farmland consolidation suitable for agricultural machinery.

As shown in **Figure 2**, one of the machine learning models—random forest (RF)—was selected to calculate the weight of indicators for assessing the suitability of FCAM in view of its successful applications in earlier studies (Ren et al., 2020). RF, as an effective prediction tool, has been widely used in research in various disciplines (Yamaç et al., 2020). RF is an ensemble learning algorithm based on a tree-based decision-making process (Taghizadeh-Mehrjardi et al., 2020; Lin et al., 2022). It is a supervised learning method that combines all tree-based results into the most appropriate model for the application (Zhang et al., 2021). RF mainly includes classification and regression techniques (Xu et al., 2019). This study mainly used RF classification technology. RF classification is a combined classification model composed of many decision tree classification models, and the parameter set is an identically distributed random vector (Belgiu and Drăguţ, 2016). Under the given independent variable, each decision tree classification model has one vote to select the optimal classification result. First, bootstrap sampling is used to extract *n* samples from the sample set as a training set. Second, *n* decision tree models are established for *n* samples to obtain *n* classification results. Finally, the RF calculated by training is used to predict the test samples and vote on each record to determine its final classification in accordance with the *n* classification results (Hayes et al., 2014). The trees are created by drawing a subset of training samples through replacement (Hengl et al., 2018). Approximately two-thirds of the samples are used to train the trees, with the remaining one-third used to test trees to estimate how well the resulting RF model performs (Aburas et al., 2019; Sekulić et al., 2020). The RF model conducts several random samplings; hence, it has a high tolerance for outliers and noise and is not prone to overfitting, and therefore possesses a high prediction accuracy (Nguyen et al., 2021).

There are a multitude of features in a dataset (Aburas et al., 2019). Selecting the features that have the most significant impact on the results, to reduce the number of features in building a model, is something that is of interest to us (Akpoti et al., 2022). In RF, the weight of features is calculated based on the Gini index. RF adopts impurity as the best division to measure the classification tree, and impurity is calculated by the index method (Su et al., 2021). Suppose that set *T* contains *k* classes of records. The Gini index is calculated by Eq. (1):


(1) $$Gini(T)=1-\sum_{j=1}^{k}p_{j}^{2}$$

where *p_j_* indicates the frequency of occurrence of *T* in category *j*.

If the set *T* is divided into *m* parts *Ti* (*i* = 1, 2,…, *m*), then, to calculate the Gini index, the reduction in the Gini index of the variable *x_i_* used to split at each split node is calculated. The Gini index for this segmentation is calculated as shown in Eq. (2):


(2) $$Gini_{split}(T)=\sum_{i=1}^{m}Gini(T_{i})N_{i}/(N)$$

where *m* indicates the number of child nodes, *N_i_* is the number of samples at descendent nodes *Ti*, and *N* is the number of samples at parent node *T*.

Generally, the value of the mean Gini index decrease for each variable over all trees in the forest is frequently used as an estimate regarding the importance of variables. Thus, the weight of features is calculated as shown in Eq. (3):


(3) $$w_{i}=\frac{D_{j}}{\sum_{j=1}^{n}D_{j}}$$

where *w_j_* is the weight, *D_j_* is the value of the mean Gini index decrease, and *n* is the number of indicators.

We investigated the pilot of the FCAM process in Gujiao City, Shanxi Province, which was completed in 2018. Therefore, we took Gujiao City as a sample area to obtain the weight of each indicator. We used ArcGIS 10.6 to extract 50 farmland attribute data points with a high degree of mechanization in the sample area and marked them as 1. Then, we extracted 50 farmland attribute data points with a low degree of mechanization in the sample area and marked them as 0. We combined the two types of data into a dataset containing 11 indicator attributes and one category attribute. The RF package in R software was used to calculate the weight of each indicator, and the results are shown in **Table 1**.

To improve the accuracy of this model, we performed a robustness analysis by using *K*-fold cross-validation. The training set is split into *k* smaller sets. The following procedure is followed for each of the *k* “folds”:

- A model is trained using *k* − 1 (9) of the folds as training data.
- The resulting model is validated on the remaining part of the data (i.e., it is used as a test set to compute a performance measure such as accuracy).

The performance measure reported by *k*-fold cross-validation is then the average of the values computed in the loop.

We used *k* = 10 in this study, and 70% of the data were utilized for training purposes and 30% of the data were used for testing. This process was repeated 10 times. Before selecting and testing new sets for the new loop, all instances in the training and testing set are randomized over the entire dataset. At the end of the 10-fold process, the average of all performance measure is calculated.

#### 2.3.3 Estimation model of the improvement potential of farmland productivity

The estimation model of the improvement potential of farmland productivity in this study was based on of the farmland productivity potential dataset of China. This dataset is based on the farmland distribution of China, soil and DEM data, and adopts the Global Agro-Ecological Zones (GAEZ) model to estimate the acquired farmland production potential in China by comprehensively considering various factors such as light, temperature, water, CO_2_ concentration, pests and diseases, agricultural climate restrictions, soil, and topography. In addition, the calculation of the improvement potential of farmland productivity has also considered the change in the area of farmland (Gónzalez et al., 2007) and the suitability of FCAM in the GAEZ model, as shown in Eqs. (4)–(6):


(4) $$P=P_{2010}\cdot (1+R_{F})\cdot (1+C_{FCAM})$$

(5) $$R_{F}=\frac{CA_{2019}-CA_{2010}}{CA_{2010}}$$

(6) $$C_{FCAM}=\frac{S_{FCAM}}{100}$$

where *P* denotes the potential productivity of farmland after the FCAM, *P*_2010_ denotes the potential productivity of farmland in 2010 using the GAEZ model, *R*_F_ denotes the changing rate of farmland area between 2010 and 2019, and *CA*_2019_ denotes the farmland area in 2019. *CA*_2010_ denotes the farmland area in 2010, *C*_FCAM_ is the coefficient of FCAM suitability, and *S*_FCAM_ is the value of suitability of FCAM (0 ≤ *S*_FCAM _≤ 100).

## 3 Results and analysis

### 3.1 Analyzing the spatial pattern of farmland

**Figure 3:**
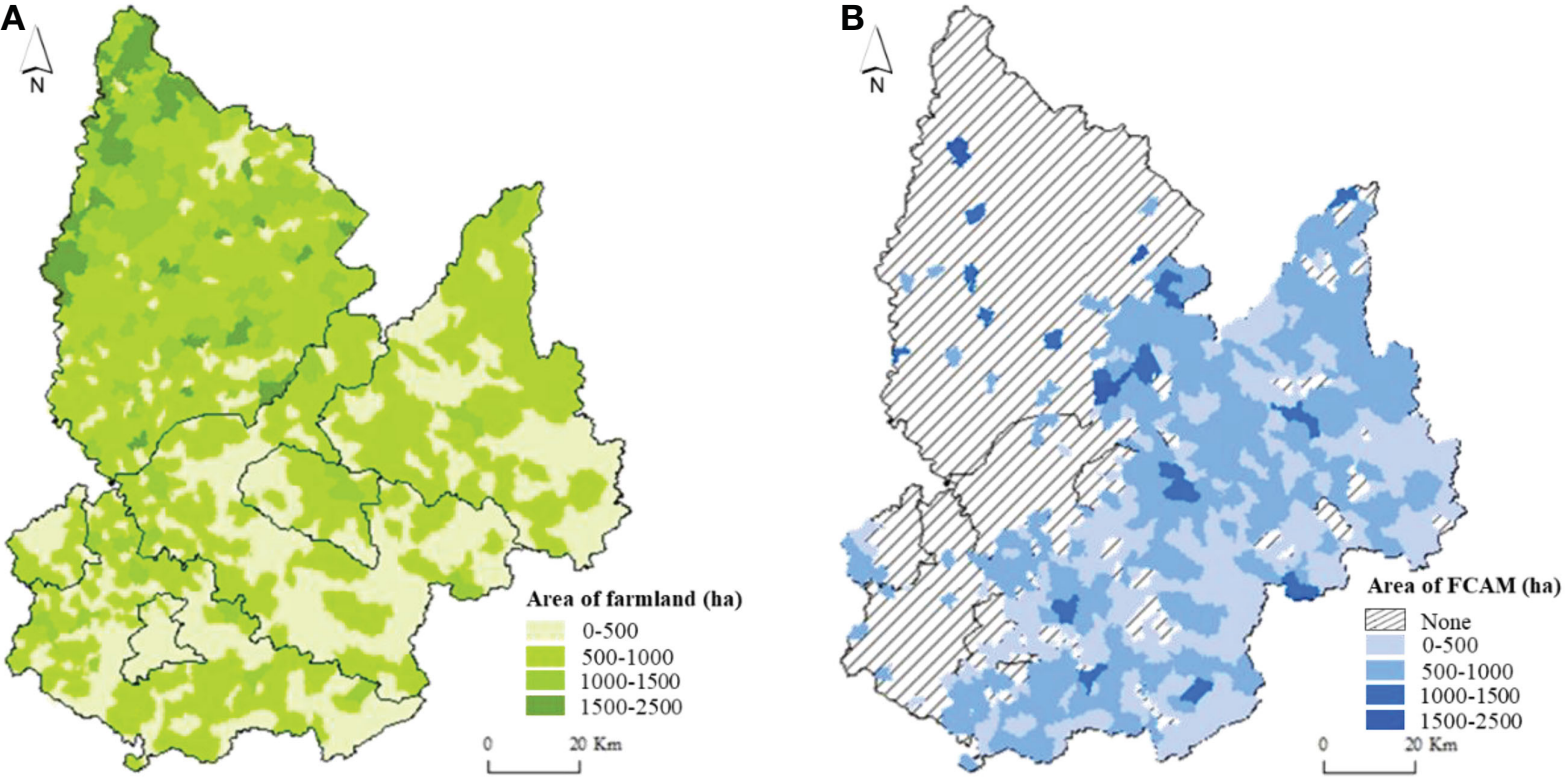
**(A)** Spatial pattern of total farmland and **(B)** area of FCAM. FCAM, farmland consolidation suitable for agricultural machinery.

In 2019, the total farmland area in Tieling City was 699.22 thousand ha, accounting for 53.80% of the city’s total area. Spatially, as shown in **Figure 3A**, we found that the area and density of farmland in the region were consistent with the “high west and low east” terrain: the area of farmland was 604.13 thousand ha in the plain areas, accounting for 86.40%. Most of the farmland was distributed in Changtu County and the whole area of Diaobingshan District, Kaiyuan County, and western Tieling County, where wide areas of plains, as well as the farmland with favorable natural conditions, were conducive to the development of agriculture and increasing large-scale farming. The area of farmland in the hilly and mountainous terrain was 95.09 thousand ha, accounting for 13.60% of the area of the city. It was mainly distributed in Xifeng County and Qinghe District, Tieling County, and eastern Kaiyuan County. The complex terrain in these regions was not conducive to agricultural production.

In accordance with international FCAM standards, FCAM schemes target slopes from 6° to 25° because farmland plots with steep slopes are not are not conducive to agricultural machinery working on farmland. However, considering the superior natural conditions of the farmland plots with slopes below 6°, all of these elements are suitable for mechanized operation. There is no need to implement FCAM schemes. In the case of farmland with slopes greater than 25°, the cost of FCAM is excessively high, resulting in a dilemma related to cost versus benefit. More importantly, the mechanized agriculture paradigm is unsuitable in these areas because the ecological conditions and soil quality of farmland restrict machinery operation.

This information has been used to obtain the area for potential implementation of FCAM in the region, as shown in **Table 2** and **Figure 3B**. Land in Tieling City that would benefit from FCAM schemes amounts to 288.47 thousand ha, accounting for 40.59% of the total farmland. It involves 458 villages, most of which are distributed in eastern Tieling City. **Table 2** shows that that there is an inverse relation between farmland distribution and slope: farmland area decreases sharply with increasing slope. Most of the farmland was distributed in the slope range of 6°–10°. Its area was 130.2 thousand ha, accounting for 45.15% of the farmland area to be consolidated and including 264 villages, most of which are in western Tieling County and northwestern Kaiyuan County.

**Table 2:** Category of farmland based on slope.

Slope range (°)	Area (thousand ha)	Percentage (%)	Number of villages	Explanation
0–6	406.21	58.09	820	No need for FCAM
6–10	130.19	18.62	264	Need for FCAM
10–15	111.03	15.88	126	
15–25	47.25	6.75	68	
> 25	4.55	0.66%	12	Unsuitable for FCAM

FCAM, farmland consolidation suitable for agricultural machinery.

### 3.2 Assessing the suitability for FCAM

Farmland suitability for FCAM was assessed based on natural resource endowment, accessibility of agricultural machinery, socioeconomic level, and ecological limitations as factors with each factor weighted in accordance with its importance. The weighting approach applied in this study is machine learning. The 10-fold cross-validation confirms the robustness of the model. From the data in **Table 3**, that the average accuracy for the weighting approach can be calculated 0.929. The validation shows that the model developed is entirely accurate. Based on the normalization, the weight of each parameter is given in **Table 1**.

**Table 3:** Result of 10-fold cross-validation of the weighting approach.

Fold	1	2	3	4	5	6	7	8	9	10
Accuracy	0.892	0.986	0.978	0.913	0.904	0.871	0.933	0.955	0.920	0.936

**Figure 4:**
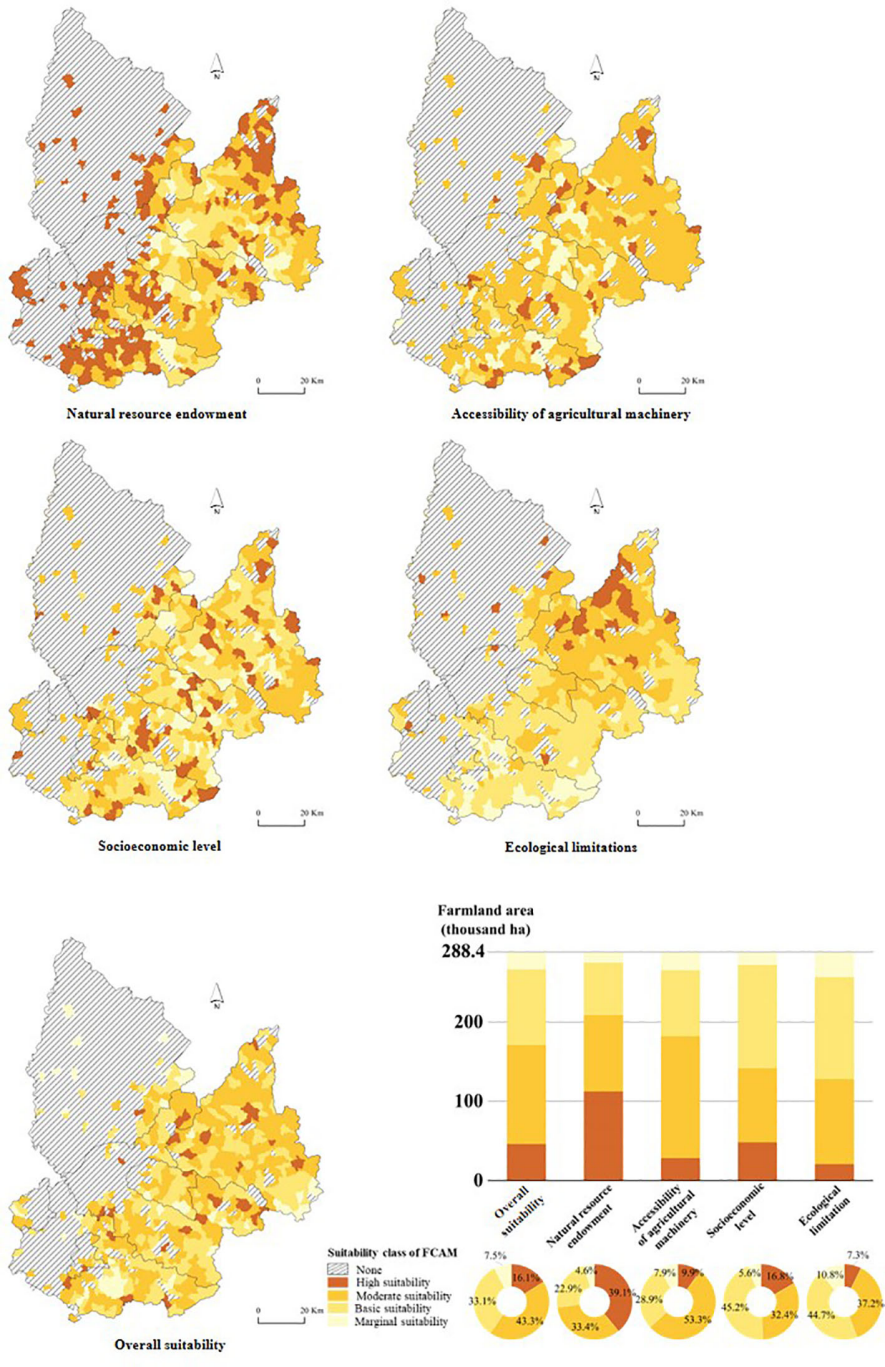
Suitability class of FCAM. FCAM, farmland consolidation suitable for agricultural machinery.

The suitability was assessed first considering each parameter separately and then taking into account their relative weights. Furthermore, the suitability of FCAM was classified into four classes (i.e., high suitability, moderate suitability, basic suitability, and marginal suitability) to reflect degrees of suitability. In this study, farmland with a slope of more than 25° was considered unsuitable for FCAM. The results of the suitability of FCAM with respect to the four parameters considered can be found in **Table 4** and **Figure 4**.

**Table 4:** Area and percentage for class of suitability of FCAM.

Classes	Natural resource endowment			Accessibility of agricultural machinery			Socioeconomic level			Ecological limitations			Comprehensive suitability		
	Area (thousand ha)	Percentage (%)	Number of villages	Area (thousand ha)	Percentage (%)	Number of villages	Area (thousand ha)	Percentage (%)	Number of villages	Area (thousand ha)	Percentage (%)	Number of villages	Area (thousand ha)	Percentage (%)	Number of villages
High suitability (75–100)	112.74	39.08	184	28.61	9.92	44	48.45	16.80	82	21.03	7.29	29	46.44	16.10	72
Moderate suitability (50–75)	96.35	33.40	133	153.79	53.31	247	93.52	32.41	135	107.25	37.18	177	124.93	43.31	194
Basic suitability (25–50)	66.03	22.89	93	83.27	28.87	134	130.36	45.19	195	128.91	44.69	196	95.49	33.10	143
Marginal suitability (0–25)	13.35	4.63	48	22.80	7.9	33	16.14	5.60	46	31.28	10.84	56	21.61	7.49	49

FCAM, farmland consolidation suitable for agricultural machinery.

#### 3.2.1 Suitability of farmland based on natural resource endowment

According to this classification, approximately 72.48% of the farmland could be categorized as either moderately or highly suitable for FCAM. The reason for this could be that the farmland was mainly distributed in wide valleys, where the superior natural resource endowment (plain terrain, large-scale plots, and favorable soil quality) makes the land suitable which facilitated the implementation of FCAM. As the main food production area in Liaoning Province, Tieling City has not only the water and heat conditions needed for crop growth, but also fertile, arable land and irrigated terrain. As a result, suitability based on natural resource endowment was relatively high.

#### 3.2.2 Suitability of farmland based on accessibility of agricultural machinery

As shown in **Figure 4B**, over 53.31% of farmland was categorized as having moderate suitability for FCAM. In addition, the suitability for accessibility of agricultural machinery in the whole region decreased from the southern to the northern areas. The farmland plots in the northern areas were deemed inferior because of their physical attributes, being scattered and typically irregular, in shape, and the poor accessibility of track roads. This could be the reason for only 9.92% of farmland showing high suitable for FCAM. Most plots are adjacent to downtown areas, a location considered economically superior because it affords greater and easier access to agricultural machinery services. In addition, the contiguity and integrity of plots in these areas were beneficial to agricultural machinery operation.

#### 3.2.3 Suitability of farmland based on socioeconomic level

In addition to the above assessment suitability criteria, socioeconomic level is also a significant determinant of the suitability of farmland for agricultural machinery operation. Demonstration of the considerable benefits that could accrue to the agricultural sector from FCAM could increase farmers’ willingness to participate in the improvement of farmland productivity. **Table 4** shows that more than 77.60% of farmland fell in the basic and moderate suitability categories. The main reason is that the continuous exodus of people from rural areas has resulted in a shortage of laborers engaged in agricultural production, and it would therefore be a considerable challenge to achieve mechanized or large-scale farming.

#### 3.2.4 Suitability of farmland based on ecological limitations

Based on the suitability classification criteria, the assessment of the suitability of farmland based on ecological limitations indicated that approximately 37.18% (107.25 thousand ha) of farmland was categorized as moderately suitable and 55.53% (160.19 thousand ha) of farmland was categorized as basically or marginally suitable for the FCAM. Although this parameter is a good indicator of ecological suitability, under FCAM schemes, since abundant forestry has resulted in high vegetation coverage, farmland consolidation is hardly suitable for agricultural machinery operations. In addition, the severe water loss and erosion caused by steep slopes adds to the ecological limitations. All things considered, suitability is generally categorized as moderate level.

#### 3.2.5 Suitability of farmland for agricultural machinery

The suitability was assessed first with respect to each parameter considered separately, and then the overall suitability of the FCAM was computed by overlaying a given level of importance or weight. Based on the suitability classification criteria, we classified the comprehensive suitability as high, moderate, basic, or marginal (see **Figure 4E**).

It was found that more than 76.41% of the farmland in the schemes fell into the basic and moderate suitability categories for agricultural machinery. Although the indicators of natural resource endowment, especially slope, are suitable for agricultural machinery, under FCAM schemes, fewer farm tracks and inferior accessibility of roads were regarded as limiting the functionality of agricultural machinery. Additionally, scarce farmland plots, as well as poor vegetation coverage, have reduced the suitability of FCAM and even caused ecological degradation. As shown in **Table 4**, 16.10% of farmland fell into the high suitability category. It was mainly distributed in Xifeng County and Kaiyuan County. The large-scale, regular plots abundant in these areas are considered to facilitate machinery operation. Moreover, a large number of laborers engaged in agricultural production (as is found in these areas) is linked to increased socioeconomic development (Makate et al., 2019). Thus, it has the potential to reduce the difficulties of implementation and high costs associated with FCAM. The area of farmland with marginal suitability was 21.61 thousand ha, which accounted for 7.49% of the total farmland area. The undulating terrain, and fragmentation of plots caused by steep slopes characterizing this area, have limited agricultural production. More importantly, considering the fragile ecological environment, what is likely to happen in these areas is soil erosion and a decline in farmland fertility. As a consequence, the inferior terrain and ecological environment makes it difficult to implement FCAM.

### 3.3 Estimation of the improvement potential of farmland productivity

**Figure 5:**
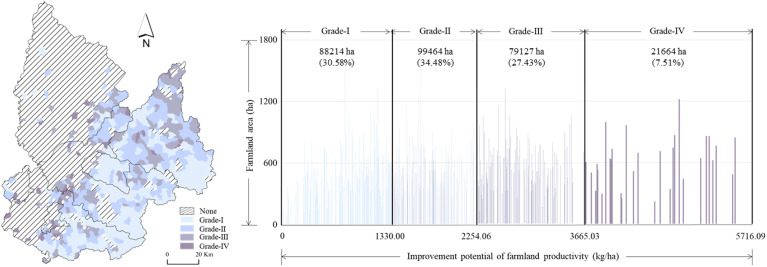
Grades of improvement potential of farmland productivity after FCAM. FCAM, farmland consolidation suitable for agricultural machinery.

According to the estimation model mentioned in section 2.3.3, farmland productivity in Tieling City ranges from 0 to 1,2203.6 kg/ha, with an average of 3,779.6 kg/ha. If FCAM were implemented in the study area, the average productivity of farmland would increase to 4,267.24 kg/ha, and the potential farmland productivity improvement could reach 720.8 kg/ha. **Figure 5** shows the estimated results for the improvement potential of farmland productivity through FCAM. We found that there were some negative values for the improvement potential of farmland productivity. The main reason for this is that the conversion of farmland to built-up land from 2009 to 2019 has resulted in a significantly decreased farmland area. The improvement in farmland productivity brought by about by FCAM cannot compensate for the reduction in productivity associated with the declining farmland area. Consequently, areas with negative farmland growth were considered unsuitable for FCAM.

We then used the Nature Breaks method to classify the improvement potential of farmland productivity according to grades, ranked from low to high (I–IV), describing the spatial patterns of the improvement potential of farmland productivity (**Figure 5**). This result indicated that the improvement potential decreased from the northeastern to the southwestern areas of land. The percentage of farmland area in grades I (0–1,330), II (1,330–2,254.06), III (2,254.06–3,665.03), and IV (3,665.03–5,716.09) was 30.58%, 34.48%, 27.43%, and 7.51%, respectively; most villages were in the moderate grades (I and II).

It was found that the improvement potential of farmland productivity mainly fell into grade II. The area of farmland classed as grade II was 99.46 thousand ha, accounting for 34.48% of the total farmland area. The original farmland productivity was relatively low because of the shortage of soil organic matter the use of traditional irrigation methods, which rely on the availability of surface water. To cope with this, the implementation of FCAM schemes could enhance farmland productivity by expanding the available farmland area, improving the soil quality, and constructing water conservancy facilities for agricultural production. Nevertheless, the shortage of labor, brought about by the majority of the economically active population being engaged in non-agricultural activities for both subsistence and commercial purposes, has to a certain extent constrained the potential improvement in farmland productivity.

The area of farmland in the low-grade group (I) was 88.21 thousand ha. Most of the farmland was distributed in the mountainous terrain, where the large area of abandoned farmland and inferior physical geography (e.g., steep slopes, barren soil, and low grain yields) and economic location ultimately reduced the potential to increase farmland productivity. The farmland area in the high-grade (IV) group was 21.66 thousand ha. Most of the farmland was distributed at the intersection of the eastern plains and the western hilly terrain. On the one hand, the advantages of physical geography and economic location (that is, naturally fertile and productive soils as well as abundant water resources in semi-hilly areas adjacent to the plain) were conducive to improved farmland productivity through FCAM. On the other hand, the local government strictly controlled the built-up land occupying farmland and attached great importance to farmland protection, which was beneficial to protecting cultivation and sustainable land development; therefore, the improvement of farmland productivity has been a natural process.

### 3.4 Zoning of FCAM schemes

**Figure 6:**
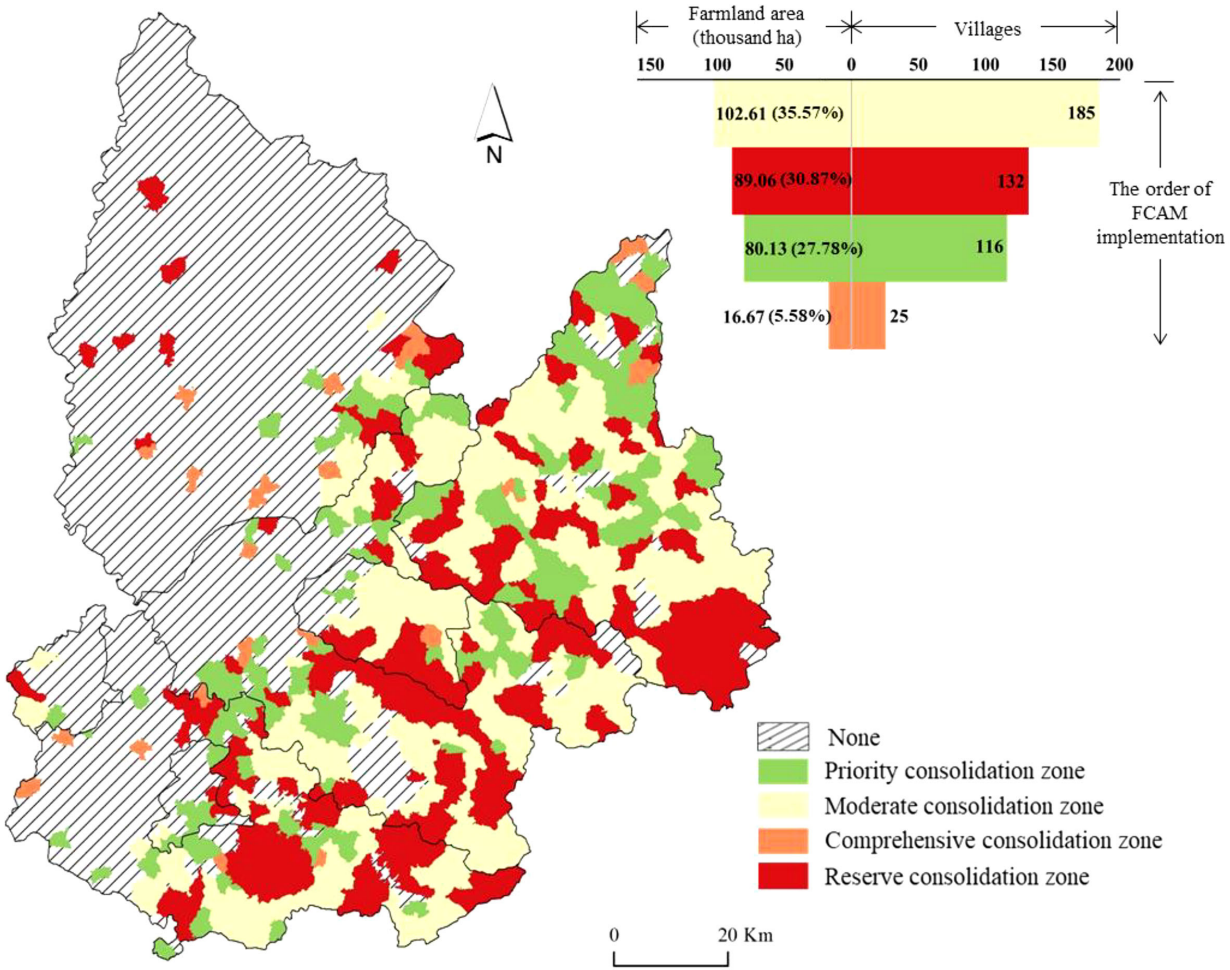
Zoning of FCAM schemes. FCAM, farmland consolidation suitable for agricultural machinery.

According to the above results, we find that the “suitability” and “improvement potential of farmland productivity” are both critical issues to consider when implementing FCAM schemes. Therefore, this study took these issues into account when categorizing the farmland used in the FCAM schemes. As shown in **Figure 6**, farmland can be categorized into four zones, i.e., the priority consolidation zone, the moderate consolidation zone, the comprehensive consolidation zone, and the reserve consolidation zone. Farmland with a relatively high class in both the suitability and improvement potential of farmland productivity should be considered for the priority consolidation zones. There was 80.13 thousand ha of farmland in this zone, accounting for 27.78% of the total farmland area. Most of this farmland is in Kaiyuan County, Xifeng County, and Tieling County, where the slope of farmland is relatively low and regular, and where there is the potential to consolidate farmland plots to achieve large-scale farming. The moderate consolidation zone comprised 102.61 thousand ha of farmland, accounting for 35.57% of the total farmland area. This region had higher farmland suitability and lower improvement potential productivity. Farmland productivity could be improved significantly because of the lower cost of implementing FCAM. Only 16.67 thousand ha (5.588%) of farmland can be categorized as a comprehensive consolidation zone; the main characteristics of this farmland were low suitability and high improvement potential. Despite the high cost of FCAM, it can bring considerable benefits to farmland productivity. In farmland with low suitability and improvement potential, FCAM cannot provide significant benefits, and these areas can be categorized as reserve consolidation zones. Farmland in this zone accounted for 30.87% of thetotal farmland area

## 4 Discussion

### 4.1 A zoning-based solution for the implementation of FCAM schemes

**Figure 7:**
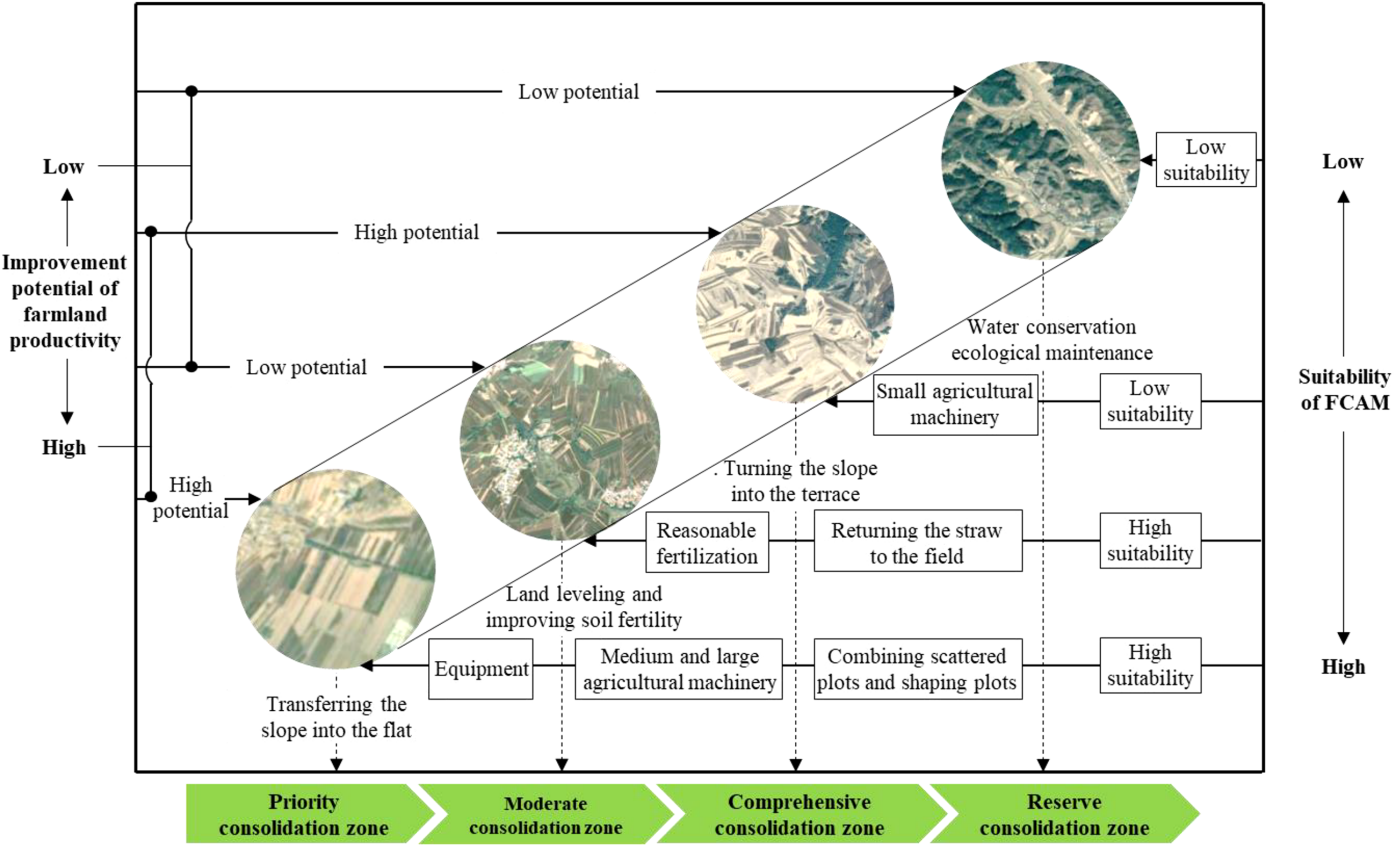
A zoning-based implementing mechanism of FCAM schemes. FCAM, farmland consolidation suitable for agricultural machinery.

According to the above considerations and results, zoning based on the “suitability for agricultural machinery” and “improvement potential of farmland productivity” has been an effective solution for implementing FCAM schemes. During the implementation process of FCAM schemes, one of the most important steps is implementation sequence. As illustrated in **Figure 7**, the implementation sequence of FCAM schemes should be consistent with these four zones (i.e., the priority consolidation zone, the moderate consolidation zone, the comprehensive consolidation zone, and the reserve consolidation zone). Moreover, differentiated policies and strategies should be proposed for each zone.

Given the gentle slope, transferring the slope into flat areas is the major measure of success of FCAM in the priority consolidation zone. In addition, considering the irregular and small-size plots, together with the complicated working path for agricultural machinery, the farmland in this zone should be arranged into large-scale and contiguous plots by combining scattered plots and reshaping plots. More importantly, as the farmland in this area has the potential to achieve large-scale farming, it is necessary to introduce some large and medium agricultural machinery and equipment to improve agricultural production efficiency. Although irregular plots and land degradation are considered to undermine the suitability of the FCAM in moderate consolidation zones, the effects of this can be alleviated by the adoption of alternative measures alternative measures, such as land leveling and improving soil fertility. In other words, we can return the straw to the field, as well as arrange reasonable fertilization to increase farmland yield and its suitability for machinery operation. At the same time, straw returning machinery and precision fertilizer application machinery can be introduced to guarantee soil quality.

Considering the restriction of the high slope of farmland in the comprehensive consolidation zone, the main measure in this area should focus on turning the slope into the terrace. In addition, the careful attention when planning the layout and design of field roads is necessary to ensure their accessibility. As farmland in this zone is mainly distributed in hilly and mountainous terrain, it is unsuitable for large and medium machinery operations. Therefore, small agricultural machinery may be a feasible alternative to sustain agricultural production. In terms of the reserve consolidation zone, there are severe ecological problems such as soil erosion and land degradation compared with other areas. It would be better, therefore, for land in this zone to be used as areserve resource in the implementation of FCAM.

### 4.2 The need for alternative strategies for achieving FCAM schemes

**Figure 8:**
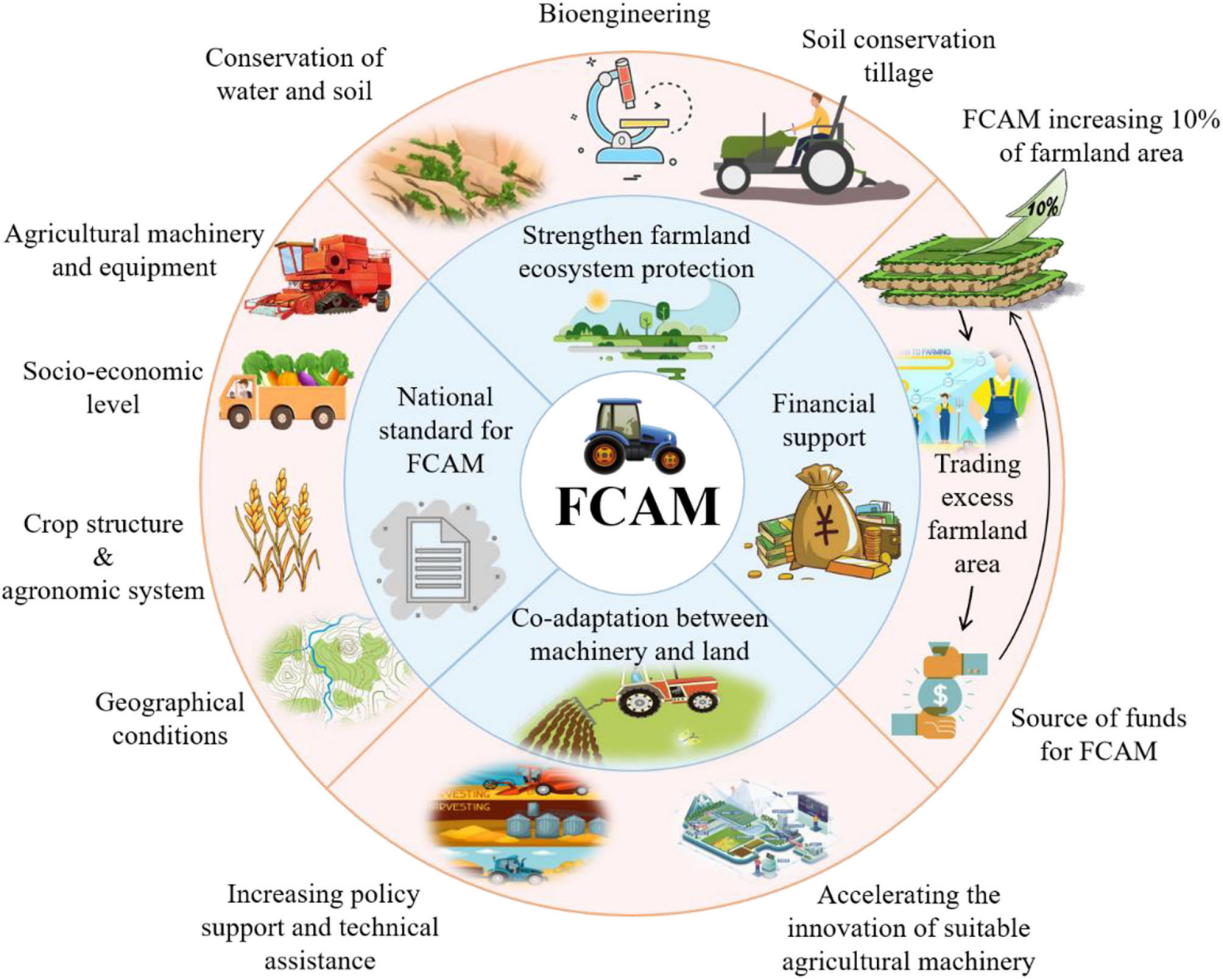
Alternative strategies for FCAM schemes. FCAM, farmland consolidation suitable for agricultural machinery.

Recently, the ecological effects of FCAM have received a great deal of attention (Huang et al., 2022), especially in hilly and mountainous areas. In fact, FCAM has positive effects on the farmland ecosystem (Zang et al., 2021). Despite this, it is necessary to strengthen farmland ecosystem protection after the implementation of FCAM. For example, the conservation of water and soil, and improving vegetation coverage by using bioengineering have been essential tasks in some areas during the process of turning slopes into terraces. In addition, soil conservation tillage helps to reduce ecological disturbance caused by agricultural machinery, for example in its increasing of soil quality. It is clear that FCAM schemes not only increase agricultural productivity but also generate ecological benefits, which could in turn lead to the achievement of sustainable agricultural production (see **Figure 8**).

There is no denying that FCAM aims to achieve co-adaptation between machinery and land. That is, R&D on agricultural machinery suitable for irregular farmland, especially in hilly and mountainous areas, should be promoted as a consequence. In particular, the government should increase policy support and technical assistance to accelerate the innovation of suitable agricultural machinery in hilly and mountain areas. Although FCAM in some provinces (such as Shanxi, Chongqing, and Sichuan) of China has been implemented for some time, there is no national standard for it. For instance, the project of “turning steep slope into terrace” did not take spatial differentiation into account, i.e., the rainfall gap is significant between the northern and southern areas of land. The terrace in the south should have been transformed into a positive slope terrace to facilitate drainage due to the high rainfall, while in the north, the terrace should have been transformed into a reverse slope terrace to reverse the water due to the low rainfall. Therefore, both national and local standards for FCAM need to be formulated in combination with many factors, including geographical conditions, socioeconomic level, crop structure, agronomic system, and agricultural machinery and equipment system, with many factors as they occur in different scenarios.

As FCAM was initiated by farmers, rather than by the government, inadequate financial support from the government has had a significant impact on the enthusiasm of farmers (Zhou and Cao, 2020). Given that the actual cost of FCAM (30 thousand yuan/ha) far exceeds the current subsidy for FCAM (22.5 thousand yuan/ha), the cost gap has forced many [farmers] to abandon FCAM. To address this and ensure the continued implementation of FCAM, the provision of more realistic subsidies should be considered by the government. According to the findings, FCAM would increase farmland area by 10%, and local governments in hilly and mountainous terrain should allow farmers to trade the excess farmland area as the source of funds for FCAM (Guan et al., 2022). In addition, governments should encourage more farmers to participate in FCAM, as this could lead to the formation of an effective and sustainable financial support system for farmers.

### 4.3 Contribution to research, limitations, and future work

As it is important for the government to guarantee an adequate food supply, FCAM schemes may be a feasible means of facilitating mechanized agriculture and could even improve agricultural productivity. This study represents a first attempt to gain a deeper understanding of the suitability and implications of FCAM schemes, which are likely to play a significant role in agricultural production in China.

We established an integrated framework to assess the suitability of FCAM, including natural resource endowment, accessibility of agricultural machinery, socioeconomic level, and ecological limitations. It is distinct from existing assessment frameworks that focus only on the physical attributes of farmland (e.g., terrain characteristics, soil quality, and availability to agricultural infrastructure) (Abubakari et al., 2016; Du et al., 2018; Keller et al., 2019), and it recognizes that FCAM, as the outcome of reciprocal relationships between farmland and agricultural machinery, is deemed important to understand both how farmland consolidation satisfies the multiple demands of the operation of agricultural machinery and how ecological limitations affect FCAM (Wiggering et al., 2006; Larsen et al., 2021). Accordingly, a systematic methodology has been introduced to assess the suitability of FCAM in hilly terrain by using a machine learning approach. In addition, compared with traditional methods that weight the indications and criteria based on a single perspective (e.g., the experts’ evaluation, AHP) (Bozdağ et al., 2016; Han et al., 2021; Kilic et al., 2022), this approach has a clear advantage in that it adjusts the weights according to dynamic changes in the environment. This can improve the effectiveness and feasibility of the weighting, enhancing the capacity of local decision-makers to determine the suitability of FCAM. The selection of assessment indicators based on ecological limitations, and the strategy obtained from the results—integrating FCAM schemes in farmland ecosystem management—are both beneficial to strengthen agroecology because they could increase agricultural productivity without damaging ecological resources. More importantly, they could be used in strategic planning to help policy-makers make location-appropriate agricultural production choices to achieve sustainable development. Consequently, in this study, the framework applied, method used, and results obtained constitute broad contributions to future research in the assessment of FCAM schemes.

However, this study has several limitations. Given the data constraints, we chose Tieling City in Liaoning Province as the case study. However, Tieling City is not representative of all hilly areas in the country; in particular, it is dissimilar to some hilly areas in southern parts of China. More effort and further analysis should be focused on a larger number of hilly areas. Second, the indicator system for assessing the suitability of FCAM focused on only four aspects, i.e., natural resource endowment, accessibility of agricultural machinery, socioeconomic level, and ecological limitations. This could be further improved by adopting indicators reflecting the integrity of agricultural infrastructure. Such an exploration would lead to a much more holistic understanding of FCAM schemes.

## 5 Conclusions

In this study, farmland suitability for the use of agricultural machinery in land consolidation schemes in hilly terrain was assessed based on four parameters, i.e., natural resource endowment, accessibility of agricultural machinery, socioeconomic level, and ecological limitations. The factors were assigned weights using a machine learning approach. Based on the assessment of the individual parameters, it is clear that the most prevalent factor making farmland area suitable for consolidation, i.e., affecting the largest proportion of farmland, is natural resource endowment. Meanwhile, low socioeconomic level and ecological limitations play a major role in reducing farmland suitability. The results of the overall suitability for FCAM showed that most of the farmland (76.41%) fell into the basic and moderate suitability categories. As highlighted in section 3.2.5, although the indicators of natural resource endowment, especially slope, could theoretically make a piece of farmland suitable for agricultural machinery, inferior accessibility of tractor roads, continuous depopulation, and ecological fragility rendered it unsuitable for agricultural machinery operation, which contributed greatly to reducing the overall suitability. Moreover, it was estimated that farmland productivity could be increased by up to 720.8 kg/ha of potential if FCAM were implemented, especially at the intersection of the eastern plains and the western hilly terrain.

Considering the importance of the “suitability” and “improvement potential of farmland productivity” for implementation of FCAM schemes, we classified land into four zones: the priority consolidation zone, the moderate consolidation zone, the comprehensive consolidation zone, and the reserve consolidation zone. This zoning has been crucial to the implementation of FCAM schemes, as the four zones constituted a useful basis for implementation sequence and differentiated strategies for FCAM schemes: consolidation should be prioritized in the case of farmland with a relatively high classification for both suitability and potential improvement of farmland productivity. Transferring the slopes into the flatter and enlarging plots have been the major measures of the success of FCAM in these areas, whereas our findings indicate that FCAM schemes in moderate consolidation zones should focus on farmland leveling and improving soil fertility. Finally, the remaining farmland can be characterized as a reserve resource because of the high cost required for its consolidation. Hence, in addition to farmland consolidation, some alternative strategies, such as strengthening farmland ecosystem protection, promoting the R&D of agricultural machinery suitable for hilly terrain, and increasing the financial support provided by the government, may contribute to the feasible implementation of FCAM schemes and the achievement of mechanized and modern agriculture.

## Data availability statement

The raw data supporting the conclusions of this article will be made available by the authors, upon reasonable request. 

## Author contributions

HY: Writing – original draft. WM: Writing – original draft, Formal analysis, Writing – review and editing. TL: Data curation, Software. WL: Conceptualization, Project administration, Supervision, Methodology All authors contributed to the article and approved the submitted version.
